# Antioxidant and Vasodilator Activity of* Ugni molinae* Turcz. (Murtilla) and Its Modulatory Mechanism in Hypotensive Response

**DOI:** 10.1155/2016/6513416

**Published:** 2016-09-04

**Authors:** Ignacio Jofré, Cesar Pezoa, Magdalena Cuevas, Erick Scheuermann, Irlan Almeida Freires, Pedro Luiz Rosalen, Severino Matias de Alencar, Fernando Romero

**Affiliations:** ^1^Center for Neurosciences and Peptides Biology, CEGIN-BIOREN-UFRO, Department of Preclinical Sciences, Faculty of Medicine, University La Frontera, Francisco Salazar 01145, 4811230 Temuco, Chile; ^2^Department of Chemical Engineering, University La Frontera, Francisco Salazar 01145, 4811230 Temuco, Chile; ^3^Department of Physiological Sciences, Piracicaba Dental School, University of Campinas, 13414-903 Piracicaba, SP, Brazil; ^4^Department of Agri-Food industry, “Luiz de Queiroz” College of Agriculture, University of São Paulo, 13418-900 Piracicaba, SP, Brazil

## Abstract

Hypertension is a systemic condition with high morbidity and mortality rates worldwide, which poses an increased risk for cardiovascular diseases. In this study, we demonstrated the antioxidant and vasodilator activity of* Ugni molinae* Turcz. (Murtilla) fruit, a berry native to Chile and proposed models to explain its modulatory mechanism in hypotensive response. Murtilla fruits were cultivated in a germplasm bank and submitted to chemical and biological analyses. The phenolic compounds gallic acid, Catechin, Quercetin-3-*β*-D-glucoside, Myricetin, Quercetin, and Kaempferol were identified. Murtilla extract did not generate toxic effects on human endothelial cells and had significant antioxidant activity against ROS production, lipid peroxidation, and superoxide anion production. Furthermore, it showed dose-dependent vasodilator activity in aortic rings in the presence of endothelium, whose hypotensive mechanism is partially mediated by nitric oxide synthase/guanylate cyclase and large-conductance calcium-dependent potassium channels. Murtilla fruits might potentially have beneficial effects on the management of cardiovascular diseases.

## 1. Introduction

Cardiovascular disorders are among the major causes of morbidity and mortality in developed and emerging countries and involve a strong public burden on treatment and therapeutic management [1]. An association between oxidative stress (OS) and the aetiopathogenesis of several cardiovascular diseases, including atherosclerosis, ischemia, stroke and hypertension, has been reported [2]. Oxidative stress resulting from an increased production of free radicals and/or failures in the antioxidant system can lead to oxidation of lipoproteins and thus development and progression of atherogenesis [3, 4], acute thrombotic events [5], and endothelial dysfunction [6], all contributing to a cardiovascular collapse.

In recent years, a number of epidemiological and pharmacological studies have suggested that consumption of fruits and vegetables is associated with a decreased risk for cardiovascular disease [6], inflammation, and cancer [7]. However, the role of individual micronutrients and phytochemicals therein is far from being understood. Fruits and vegetables contain a large variety of biologically active elements such as ascorbic acid [8], folate [9], potassium, and soluble fibers [10]. These aspects suggest that their consumption can positively modify markers of cardiovascular disease, such as blood pressure and cholesterol [11]. In addition, phenolic compounds biologically active, such as flavonols, phenolic acid, anthocyanins, and procyanidins are found in high concentrations in several fruits [12].

Berries are considered another rich source of antioxidant molecules able to decrease blood pressure, increase HDL levels, and stimulate platelet function [13]. Murtilla or myrtle (*Ugni molinae* Turcz.) is a native Chilean species belonging to the Myrtaceae family rich in phenolic components, mainly flavonoids [14]. Studies on the chemical composition of Murtilla leaves and fruits indicate the presence of flavan-3-oles and a variety of flavonoids (Catechin, Myricetin, kaempferol, and Quercetin) [15, 16]. In this study, we demonstrated the antioxidant and vasodilator activity of the aqueous extract from the fruits of* Ugni molinae* Turcz. (Murtilla) and proposed models to explain its modulatory mechanism in hypotensive response.

## 2. Materials and Methods

### 2.1. Chemicals

Acetylcholine, atropine, modified Tyrode's Salt Solution, and N*ω*-nitro-L-arginine methyl ester hydrochloride were acquired from Sigma-Aldrich Chemical Co. (St. Louis, MO, USA); NaCl, KCl, CaCl_2_·6H_2_O, MgCl_2_·6H_2_O, NaHCO_3_, NaH_2_PO_4_, and C_6_H_12_O_6_·H_2_O were purchased from Merck (Darmstadt, Germany). Phenylephrine was acquired from AcrosOrganics (Geel, Belgium). The blockers CNCKAPETALCARRCQQH (apamin), XFTNVSCTTSKECWSVCQRLHNTSRGKCMNKKCRCYS (charybdotoxin), 1H-[1,2,4]oxadiazolo[4,3-a]quinoxalin-1-one (ODQ), (9S,10R,12R)-2,3,9,10,11,12-hexahydro-10-methoxy-2,9-dimethyl-1-oxo-9,12-epoxy-1H-diindolo[1,2,3-fg:3′,2′,1′-kl]pyrrolo[3,4-i][1,6]benzodiazocine-10-carboxylic acid, and methyl ester (KT5823) were obtained from Tocris Bioscience (Bristol, UK).

### 2.2. Plant Material and Extraction Procedure

Murtilla fruits (*Ugni molinae* Turcz.), genotype 14-4, were cultivated in the germplasm bank of the experimental station of the Agricultural Research Institute INIA-Carillanca, Puerto Saavedra, La Araucanía, Chile. Freshly harvested fruits collected in April 2012 presented a moisture content of 78.2% (wet basis) and 19.7°Brix. Six grams of the fruits was ground in a mortar and transferred to a bottle containing 20 mL of prewarmed (30°C) distilled water. The mixture was shaken in an incubator (GFL-3032, Germany) at 170 rpm, 30°C, for 20 min and vacuum-filtered (Whatman #1 filter paper). The aqueous extract was protected from light and oxygen under refrigeration and used for determination of total phenolic content and high-performance liquid chromatography (HPLC) analysis. For HPLC analysis, the original extract was concentrated to dryness using a rotary evaporator (Büchi R-210, Germany) at 140 rpm, 30°C and then redissolved in 5 mL of methanol : formic acid (99 : 1, v/v).

### 2.3. Chemical Analysis

#### 2.3.1. Determination of Total Phenolic Content

The total phenolic content was determined using the Folin-Ciocalteu method as described by [17]. Aqueous Murtilla fruit extract (40 *μ*L) was mixed with distilled water (3.16 mL) in a test tube and then 200 *μ*L of Folin-Ciocalteu reagent was added. After 5 min at 20°C, 600 *μ*L of 20% Na_2_CO_3_ was added to the reaction mixture, which was maintained at 20°C for 120 min in darkness. The absorbance was measured at 765 nm using a spectrophotometer (SpectronicGenesys 5, Sweden), and the results were expressed as *μ*g of gallic acid equivalent (GAE) per mL of aqueous extract.

#### 2.3.2. Identification of Phenolic Compounds by HPLC

Phenolic compounds were identified using the Merck Hitachi High-Performance Liquid Chromatography system (LaChrom, Tokyo, Japan) coupled to an L-7100 pump and L-4250 UV-VIS detector. A 5 *μ*m C-18 RP Inertsil ODS-3 column (GL Sciences Inc, Tokyo, Japan) with a 250 mm × 4.60 mm i.d. was used and maintained at 25°C. The sample extract was filtered through a 0.45 *μ*m filter, and 20 *μ*L was injected for the analysis of polyphenols. The identification of compounds was confirmed both by comparison of their retention time with pure standards and by coinjection. A linear gradient solvent system consisting of 1% formic acid (A) and acetonitrile (B) was used at a flow rate of 1 mL/min as follows: 0–2 min, 100% A; 2–15 min, 80% A/20% B; 15–20 min, 70% A/30% B; 20–30 min, 40% A/60% B; and 30–35 min, 100% A. Phenolic compounds were detected at 280 nm [17].

### 2.4. Cytotoxic Effects of the Fruit Extract on HUVEC-C Cells

The cytotoxic effects of Murtilla fruit extract were determined on Human Umbilical Vein Endothelial Cells (HUVEC) ATCC® CRL-1730. Cells were maintained in Dulbecco's Modified Eagle's Medium (DMEM) high glucose (Gibco, Grand Island, NY) supplemented with 10% Bovine Fetal Serum (BFS) and 1% antibiotic (penicillin/streptomycin) (Hyclone, Salt Lake City, UT) at 37°C, 5% CO_2_. Cell viability upon treatment with the fruit extract was assessed through the MTT method described by Mossman [18] with modifications. Briefly, cells were harvested and seeded onto 96-well microtiter plates (Thermo Scientific, Rockford, USA) containing DMEM plus BFS at a density of 5 × 10^4^ cells mL^−1^ and allowed to adhere for 24 h. After that, the adhered cells were exposed to Murtilla extract at concentrations ranging from 0.001 to 44 *μ*gGAE/mL. After 24 h incubation, viability was determined by adding 5 *μ*g/mL of MTT solution, followed by incubation at 37°C for 2 h. Cells were washed with HBSS 1x (Hanks's Buffer Salt Solution, Hyclone, Salt Lake City, UT), DMSO was added, and the plate was kept at room temperature for 2 h. Final DMSO concentration did not affect cell viability. The succinate dehydrogenase activity was evaluated by color changes and the absorbance was measured at 514 nm using a Biotech Synergy-HT multiscan photometer.

### 2.5. Antioxidant Assays

The antioxidant activity of Murtilla fruit extract was determined by the luminescence reaction of luminol [5-amino-2,3-dihydro-1,4-phthalazinedione] (Sigma); membrane lipid peroxidation using Bodipy C-11 sensor (Invitrogen); and intracellular superoxide anion production using dihydroethidium (DHE, Invitrogen), a sensor of superoxide anion (O_2_^−^), using procedures described by the manufacturers.

#### 2.5.1. Luminol-Chemiluminescence Assay

This method can measure oxidant molecules that activate the luminol dye from a reduced to an oxidized form, usually by using a ferrous donor catalyst, such as potassium ferricyanide. Briefly, 50,000 cells/mL were incubated with H_2_O_2_ (200 *μ*M) for 2 h in high glucose DMEM. Increasing concentrations of extract (0.1 to 44 *μ*gGAE/mL) were added for 30 min, followed by addition of 600 *μ*M luminol and incubation for 15 min at room temperature. Luminescence was measured in a luminometer (Luminoskan, Thermo Scientific) with 1000 ms integration. The luminescence intensity (represented in %) was compared with that of a control without antioxidant treatment [19].

#### 2.5.2. Membrane Lipid Peroxidation

For the lipid peroxidation assay, 50,000 cells/mL were incubated in DMEM with 100 *μ*M H_2_O_2_ as oxidizing agent for 1 hour at 37°C. The cells were exposed to increasing concentrations of Murtilla extract (0.1 to 4.6 *μ*gGAE/mL) for 30 min and then washed with PBS. Bodipy C-11 (1 *μ*M) was added to the solution and the plate was incubated for 15 min at 37°C. The cells were washed twice and the absorbance was measured in microplate reader (Biotek Synergy HT) using the recommended spectra (488/585 for oxidized fraction and 488/595 for nonoxidized fraction, 35% sensibility). All treatments were compared with their corresponding control. The images were obtained through confocal microscopy (Olympus Fluoview 1000) [20].

#### 2.5.3. Intracellular Superoxide Anion Production

Dihydroethidium (DHE) was used in the superoxide anion assay. A total of 50,000 cells/mL were incubated for 24 h in DMEM medium. Then 100 *μ*M H_2_O_2_ and increasing concentrations of Murtilla extract (0.1 to 4.1 *μ*gGAE/mL) were added for 30 min. One micromolar of DHE was added per well and the plate was incubated for 15 min at 37°C and then washed twice. The fluorescence intensity was measured using a microplate reader under the spectra 488/595, with 35% sensibility. All treatments were compared with a control and normalized considering a basal anion production. The corresponding images were obtained through confocal microscopy (Olympus Fluoview 1000) [21, 22].

### 2.6. Vasodilator Effects of the Fruit Extract in Aortic Rings

#### 2.6.1. Ethical Considerations

All experiments were performed in accordance with the ethical principles of the National Institutes of Health (NIH), Bioethics and Biosecurity Manual of FONDECYT, after approval by an Institutional Review Board of Universidad de La Frontera, Chile.

#### 2.6.2. Experimental Design

Three normotensive male rats (Sprague Dawley) weighing 200 g were used for the vasodilation experiments. The animals were sacrificed and a thoracotomy procedure was performed to extract their aortic tissues. Dissection was carefully done to extract the connective tissues without damaging smooth muscle and endothelial structures. The isolated tissue was maintained in Tyrode modified solution under constant oxygenation (95% O_2_ and 5% CO_2_) at 37°C [we will name the Tyrode medium and oxygenation conditions and temperature as TYRO37 henceforth]. Assays without endothelial tissue were also performed. In these cases, the aorta was dissected in rings of 3 to 5 mm approximately and mounted on a silver stem in isolated organ bath (LSI, Letica Scientific Instrument) in TYRO37 conditions for 10 min. The mounted rings were subjected to 1 gf tension and stabilized for 30 min. The data were measured and processed on Powerlab version 5.5 (ADinstruments).

The contractile response was stimulated using 60 mM KCl for 10 min and serial washes under TYRO37 conditions. In order to identify the integrity of aortic tissues and endothelium, 0.5 *μ*M phenylephrine was applied and maintained before reaching the contractile plateau, and then 10 *μ*M acetylcholine was added to the solution, evidencing a nitric oxide- (NO-) dependent vasodilation. The tissues were washed with TYRO37 and 0.5 *μ*M phenylephrine was added until reaching a plateau. Then different concentrations of Murtilla (0.001 to 44 *μ*gGAE/mL) were added to the system, with and without endothelium.

In order to elucidate the mechanisms involved in these experiments, the effects of Murtilla extract on the aortic rings (*n* = 8) were evaluated in the presence of selective inhibitors. So, aortic rings were preincubated for 20 min with 1 *μ*M apamin (selective inhibitor of K^+^ channel Ca^2+^-dependent of low conductance); 100 *μ*M charybdotoxin (specific inhibitor of K^+^ channel Ca^2+^-dependent of high conductance); 2 *μ*M KT5823 (PKG inhibitor); 1 *μ*M L-NAME (eNOS and iNOS inhibitor); or 5 *μ*M ODQ (selective irreversible inhibitor of guanylate cyclase competitive with NO) and then exposed to 0.5 *μ*M phenylephrine until reaching the plateau stage. Finally, different concentrations of Murtilla extract were added to the system (0.001 to 44 *μ*gGAE/mL), and the effects were measured. In all assays, the tension was normalized to a basal level (0% of contraction) and 100% of contraction induced by phenylephrine.

### 2.7. Statistical Analysis

The antioxidant assays were performed in quintuplicate of three independent experiments. In the cardiovascular assays, a total of three rats were used to obtain the tissues, and experiments were performed in quadruplicate. To determine the effective concentrations of the extract in antioxidant and cardiovascular assays, a nonlinear regression model (dose-response) was used on Graphpad Prism 5.0 (San Diego, CA, USA). Images in this study were generated by confocal microscopy (Olympus Fluoview 1000) and processed on Image J software (NIH, USA).

## 3. Results and Discussion

### 3.1. Chemical Characterization of Murtilla Fruit Extract

The total phenolic content in the aqueous extract of Murtilla was 88.7 mg gallic acid equivalent/L, which is lower than that reported for aqueous Murtilla fruit extracts from three locations in Chile with diverse climatic conditions [15]. The phenolic compounds identified by HPLC analysis in Murtilla fruit extract were gallic acid, Catechin, Quercetin-3-*β*-D-glucoside, Myricetin, Quercetin, and Kaempferol at different concentrations (Table 1). These compounds have been commonly found in Murtilla fruit and leaves [15–17, 23] particularly the flavonol Catechin, which was the most abundant constituent in our sample (2.696 *μ*gGAE/mL). Recent clinical and experimental data demonstrated the anti-inflammatory potential of green tea Catechins for the management of cardiovascular diseases [24], particularly because of their potent antioxidant properties [25]; therefore, Catechins have been considered valuable chemical markers for antioxidant activity in naturally occurring agents. Gallic acid, Myricetin, Quercetin, Quercetin-3-*β*-D-glucoside, and Kaempferol were also found in Murtilla fruit extract at concentrations ranging from 0.009 to 0.140 *μ*gGAE/mL. Some of these compounds have been reported in other types of endemic berries in Chile (*Vaccinium corymbosum, Berberis microphylla, Luma chequen, Luma apiculata*, and* Amomyrtus meli*), in addition to* Ugni molinae* Turcz. (Murtilla). The fruit extracts of these berries were shown to have antioxidant activity based on their DPPH radical bleaching activity and ferric reducing antioxidant power (FRAP) and high ORAC activity [26–28].

### 3.2. Cytotoxicity on Endothelial Cells and Antioxidant Properties

**Figure 1:**
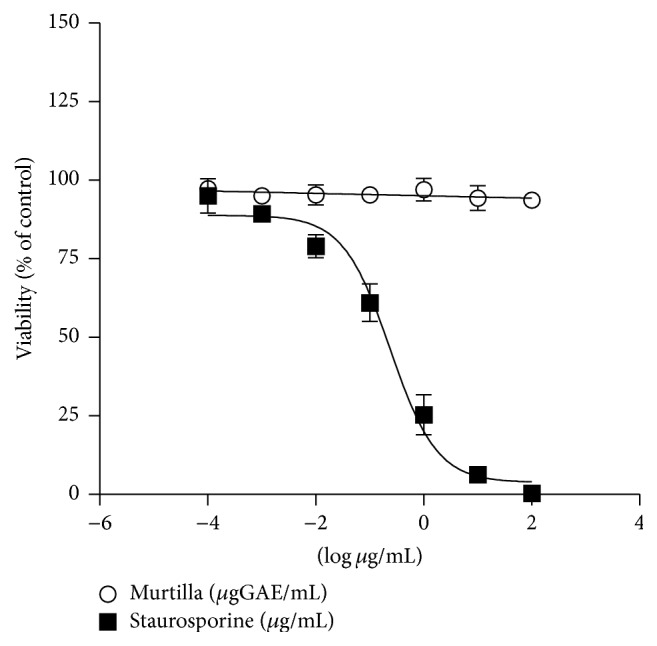
Cytotoxic effects of Murtilla fruit extract on HUVEC-C cells. Analysis of cell viability in endothelial cells incubated with Murtilla fruit extract (0.001 to 44 *μ*gGAE/mL) (circles) or staurosporine (square). The assay was performed in quintuplicate using the MTT colorimetric method. Dose-response curves were plotted using a nonlinear regression model.

The results show that the tested concentrations of Murtilla extract (0.001 to 44 *μ*gGAE/mL) did not generate toxic effects on HUVEC-C cells, as there was only 2% death at the highest concentration of the extract. Staurosporine, a protein kinase inhibitor, was used as a positive control and markedly affected cells with dose-dependent effects (Figure 1).

**Figure 2:**
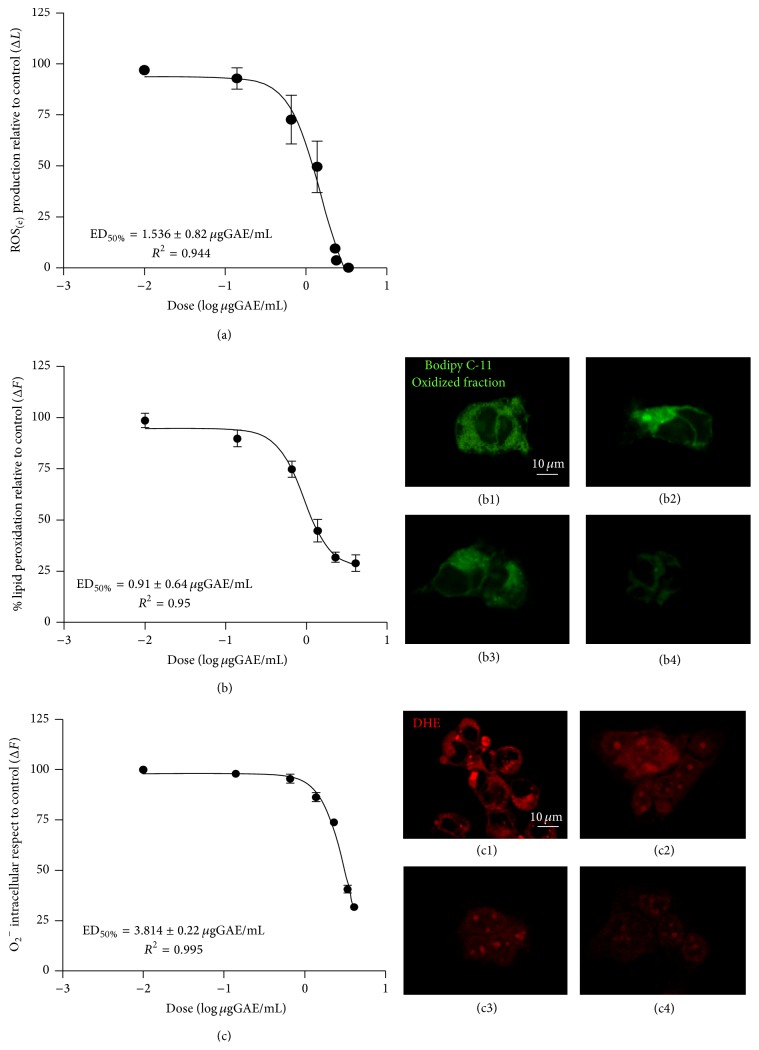
Antioxidant analysis of Murtilla in endothelial cells. (a) Effects of increasing concentrations of Murtilla (0.01–44 *μ*gGAE/mL) upon ROS production, measured by the chemiluminescent reaction of luminol. (b) Effects of increasing concentrations of Murtilla (0.01–44 *μ*gGAE/mL) on reduction of lipid peroxidation induced by H_2_O_2_, measured by BODIPY C-11 dye; (b1) control; (b2) 0.01 *μ*gGAE/mL; (b3) 1 *μ*gGAE/mL; and (b4) 44 *μ*gGAE/mL: the oxidized fraction is shown. (c) Effects of increasing concentrations of Murtilla (0.01–44 *μ*gGAE/mL) on reduction of intracellular superoxide anion (O_2_^−^) produced by H_2_O_2_, measured by dihydroethidium dye; (c1) control; (c2) 0.01 *μ*gGAE/mL; (c3) 1 *μ*gGAE/mL; (c4) and 44 *μ*gGAE/mL: the oxidized fraction is shown. All assays were performed in quintuplicate of three independent experiments, and dose-response curves were plotted using a nonlinear regression model.

The antioxidant activity of Murtilla fruit extract upon extracellular and intracellular oxidative stress was determined by different methods. The luminol-chemiluminescence assay indicated a significant dose-response effect of the extract against ROS production, with ED_50%_ of 1.53 ± 0.82 *μ*gGAE/mL in 1 hour. At the concentration of 4.1 *μ*gGAE/mL Murtilla extract inhibited almost 100% of ROS production (Figure 2(a)). As ROS are considered a group of destructive molecules produced in the cell through metabolism of oxygen [29], their inhibition is highly desirable.

Membrane lipid peroxidation was reduced upon exposure to Murtilla extract, with DE_50%_ of 0.091 ± 0.4 *μ*gGAE/mL (Figures 2(b) and 2(b1)–2(b4)). Some studies [30] have shown that lipid peroxidation is one of the processes associated with cardiovascular diseases, such as hypertension and arteriosclerosis, so the use of nonenzymatic antioxidants (e.g., nutritional supplements) could generate a preventive effect on the endothelium and smooth muscle cells. Our experiments showed that Murtilla extract can reverse such oxidative process. H_2_O_2_ by Fenton's reaction generates hydroxyl radicals in the presence of metals such as Fe^++^ playing a key role in lipid peroxidation, because combination with unsaturated lips (process of oxidative initiation) generates lipid oxidation and subsequently propagation of oxidation to unoxidized unsaturated lipids [31]. Our experiments showed that Murtilla extract can limit the generation of ROS and exert protective scavenging effects, in addition to limiting the propagation of peroxidation in the membrane.

Finally, the cells exposed to Murtilla extract showed reduced intracellular superoxide anion (O_2_^−^) production by the dye DHE with dose-dependent effects (ED_50%_ of 3.814 ± 0.22 *μ*gGAE/mL), as seen in Figures 2(c) and 2(c1)–2(c4). Intracellular OS, specifically in relation to the production of superoxide anion (and other groups), is associated with proinflammatory processes such as ischemia/reperfusion injury, diabetes, and obesity [32–34]. When there is an increase in the amount of H_2_O_2_ in the cell, the enzyme catalase converts H_2_O_2_ into water and molecular oxygen (mostly reactive) until saturation, a cyclical process that produces superoxide reactivity and leads to the accumulation of superoxide anion in important cell compartments, such as the nucleus, and regulatory biochemical centers. This process is associated with the loss of endothelial function and hence with endogenous regulation processes in vascular tension [35, 36]. Our data indicate that Murtilla extract can effectively reduce the superoxide anion production at low concentrations. This process is not yet elucidated for most nonenzymatic antioxidants, although some molecules of great importance such as Quercetin, Kaempferol, and gallic acid have shown a great antioxidant potential. Because our sample has different molecules it was not possible to determine the mechanism by which the transport is mediated in the membrane.

**Figure 3:**
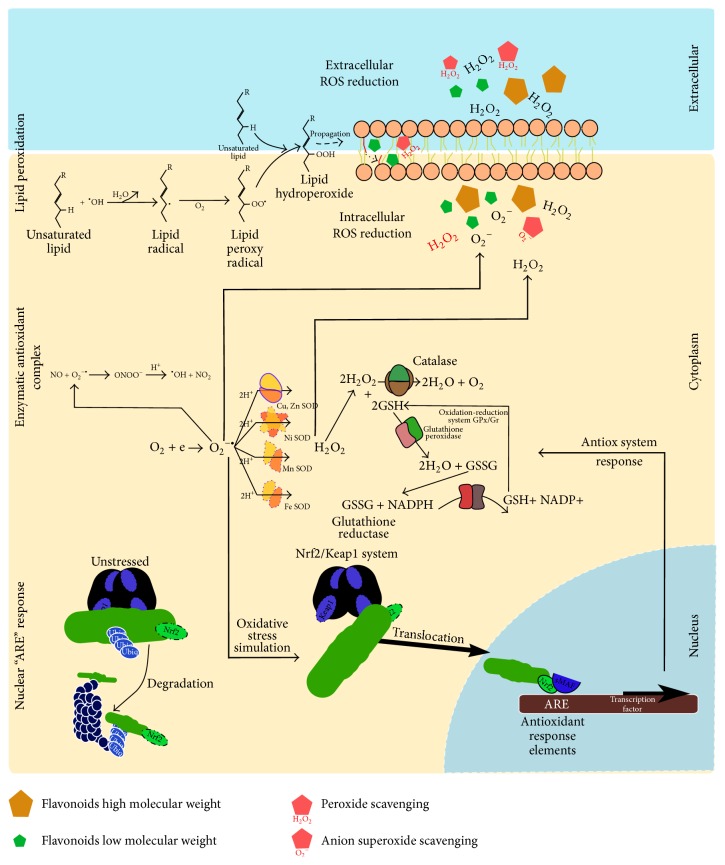
Possible antioxidant response of flavonoids compounds combined with Nrf2 pathway. Extracellular reduction of oxidative stress scavenging by flavonoids compound reducing an oxidative damage by H_2_O_2_, decreasing the concentration potentially dangerous to membrane and intracellular milieu. The reduction of intracellular oxidative stress can be mediated by Keap1/Nrf2 pathway by the activation of antioxidant response element (ARE) through the Nrf2 phosphorylated and translocated to nucleus; this activates the transcription factor to increase concentration of antioxidant complex, reducing ROS by enzymatic system. In this sense, the flavonoids can act as scavenger of ROS, enhancing the intracellular ROS balance and reducing the lipid peroxidation and the propagative effect.

Altogether, the results show a protective effect of Murtilla against OS, which is a topic of major interest in current days, because OS has been associated with the aetiopathogenesis of several chronic diseases, including arthritis, cancer, diabetes, atherosclerosis, ischemia, failures in immunity, and endocrine functions, among others [2]. In this sense, the regulation of OS is differentiated, some studies showed that the berry constituents, Quercetin, Kaempferol, and pterostilbene, synergistically attenuate the OS by involvement of the nuclear factor (erythroid-derived 2)-like 2 signaling pathway (Nrf2). This system Nrf2 regulates antioxidative stress enzymes and phase II drug metabolizing/detoxifying enzymes by binding to antioxidant response element (ARE) (Figure 3). The authors found that the combined treatment significantly induced ARE and increased the mRNA and protein expression of Nrf2-regulated genes [37]. Under basal conditions, Nrf2 is retained in the cytosol by binding to the cytoskeletal protein Keap1. Upon exposure to oxidative stress or other ARE activators, Nrf2 is released from Keap1 and translocates to the nucleus, where it can bind to the ARE, leading to the expression of antioxidant and phase II enzymes that protect the cell from oxidative damage [38, 39]. Some flavonoids as Quercetin (constituent of Murtilla extract) has been studied for present influence in the mechanism of modulation of Nrf2 and Glutathione-related enzymes as glutamylcysteine-synthetase, Glutathione-peroxidase, and Glutathione-reductase by the p38-MAPK pathway in HepG2 cells [40], while, in* in vivo* conditions, it was demonstrated in Sprague-Dawley rats that the administration of Catechin prior to the administration of Ketoprofen successfully inhibited oxidative damage by upregulating Nrf2 [41]. In our experiment we determine the antioxidant capacity detecting some important oxidizing agents as hydrogen peroxide and superoxide anion focusing on reduce the oxidative stress. This can conduce different forms to control of oxidative stress by Murtilla extract, probably, acting as radical scavenging improving the antioxidant response, as well as modulating an intrinsically pathway to stimulate an activation of ARE to increase the antioxidant enzymes. We propose more experiment to determine the mechanism of flavonoids to stimulate the Nrf2 pathway and quantify the enzymes implicated in this process, even more when these compounds are applied altogether.

### 3.3. Vasodilator Effects in Aortic Rings

**Figure 4:**
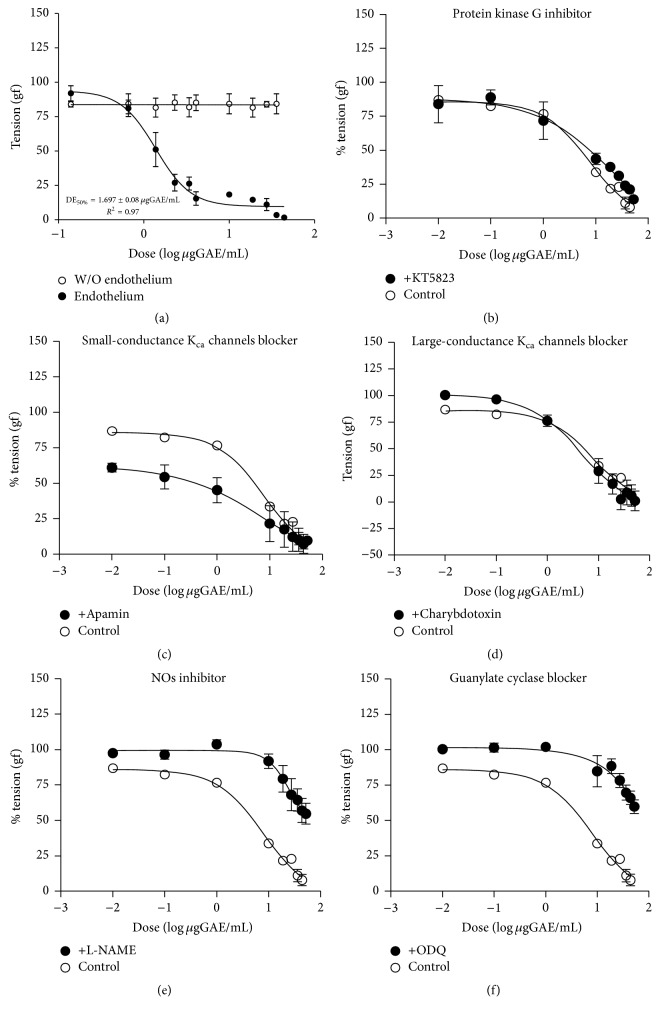
Vasodilator effects of Murtilla on isolated aortic rings. (a) Dose-dependent effects of Murtilla fruit extract (0.1 to 44 *μ*gGAE/mL) upon aortic tissues with intact endothelium (black circles) or without endothelium (empty circles). Aortic rings with endothelium were exposed to a protein kinase G inhibitor ((b) black circles); small-conductance K_ca_ channel blockers ((c) black circles); large-conductance K_ca_ channels blocker ((d) black circles); nitric oxide synthase inhibitor ((e) black circles); and guanylate cyclase blocker ((f) black circles) for 20 minutes and then treated with 0.1 to 44 *μ*gGAE/mL Murtilla extract to induce vasorelaxation. All treatments were compared with their controls without blockers (white circles). All assays were performed in quintuplicate of three independent experiments, and dose-response curves were plotted using a nonlinear regression model.

The vasodilator properties of Murtilla extract were tested using aortic rings with or without endothelial tissue. Murtilla fruit extract showed dose-dependent vasodilator activity in the presence of endothelial tissue with ED_50%_ of 1.69 ± 0.08 *μ*gGAE/mL, while no vascular activity was observed in the tissue without endothelium, as seen in Figure 4(a). Selective inhibitors were used to elucidate possible mechanisms of action of Murtilla fruit extract. Its vasodilator effects were not blocked in the presence of a protein kinase G inhibitor (Figure 4(b)) and small- and large-conductance calcium-dependent potassium channel (K_ca_) inhibitors (Figures 4(c) and 4(d), resp.). On the other hand, vascular reactivity was inhibited in the presence of eNOS (Figure 4(e)) and guanylate cyclase (Figure 4(f)) blockers.

In recent years, the intake of fruits has been shown to have an impact on the incidence and markers of cardiovascular diseases [7], as well as their active components. Some flavonoids as Catechin have been demonstrated as important antioxidant which can reduce the oxidative stress associated with endothelial dysfunction which improves antioxidant defense [42] scavenging ROS and chelating redox-active transition of metal ions [43] and, besides that, have the ability to prevent atherosclerosis, hypertension ischemic heart diseases, and other cardiovascular disorders enhancing the vascular integrity and regulating the blood pressure [44]. About this, the cardiovascular effect of flavonoids is not new; for example, the activity of Quercetin and Myricetin has distinct signaling mechanism; Quercetin induced biphasic inotropic and lusitropic effect, positive at lower concentrations and negative at higher concentrations, while Myricetin elicits coronary dilation without affecting contractility and relaxation [45].

In order to determine whether Murtilla extract had vasodilator effects, we incubated rat aortic rings previously contracted with PE and evaluated the tension generated in the presence of Murtilla extract at increasing concentrations. The extract produced a dose-dependent decrease in the tension generated by the aorta. It has been demonstrated that the regulation of vascular tone depends on nitric oxide production by endothelial cells [46] and hyperpolarization of smooth muscle cells via activation of large, intermediate, and short conductance calcium-dependent potassium channels. In our model, the elimination of the endothelial cell layer from the aorta rings reversed the vasorelaxant effect induced by Murtilla extract (Figure 4(a)). These data suggest that the vasolidator mechanism seems to be associated with NO production by endothelial NOS instead of switching the polarity of smooth muscle cell membrane. To test this hypothesis, we did the same experiment using an eNOS selective blocker (L-NAME). We found that, in the presence of low or high concentrations of Murtilla extract, the eNOS blocker partially inhibited the effect mediated by the extract (Figure 4(e)), suggesting that the effect may be related to an endothelial component and endothelial-dependent smooth muscle hyperpolarization. NO diffuses from the endothelial cell into the smooth muscle cell, where it stimulates cGMP production and activation of PKG. The conversion of GTP into cGMP and activation of PKG were also blocked in this study using ODQ and KT5823, respectively. The data showed that the vasorelaxant effects of Murtilla were partially reversed in the presence of ODQ (Figure 4(f)), whereas no changes were observed when using PKG inhibitor (Figure 4(b)).

Another mechanism that has been proposed to explain vasodilation is endothelium hyperpolarization via opening calcium-dependent potassium channels (small-conductance calcium-activated potassium channel, SK; intermediate-conductance calcium-activated potassium channel, IK; and large-conductance calcium-activated potassium channel, LK); the first two are expressed on the endothelium, while the last one is expressed in muscle cells [47]. The blocking of SK channels with apamin produced a drop in the tension generated in the aortic rings beyond the values found in the presence of increasing doses of Murtilla, suggesting that the molecular targets of Murtilla extract may be not only the NO-GCs signaling cascade, but also IK and LK channels. This was demonstrated by blocking these channels with charybdotoxin, a LK channel inhibitor; the data showed no significant differences in % tension when treated either with the extract or with charybdotoxin (Figure 4(d)).

**Figure 5:**
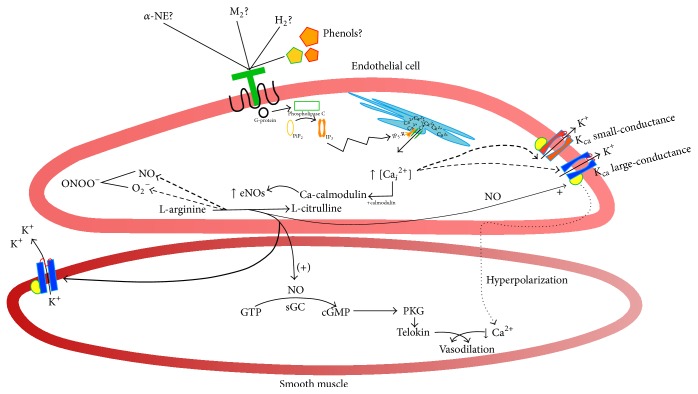
Proposed model for the hypotensive mechanisms of Murtilla fruit extract. The phenolic compounds in Murtilla activate the vasorelaxation response partially by nitric oxide synthase (NOS)/guanylate cyclase when the tissue is exposed to low concentrations of the extract, while at high concentrations the effect is unspecific or vasodilation could be regulated by an alternative pathway. However, when the smooth muscle cell is blocked using a protein kinase G inhibitor, the effect is no longer dependent on eNOS; otherwise it is apparently modulated by an alternative pathway, as we did not observe an inhibitory effect upon stimulation with the extract. In the smooth muscle cell, there could be a hyperpolarization phenomenon leading to vasorelaxation when small-conductance K_ca_ channels are inhibited, probably because large-conductance channels remain active and enhance the vasodilator effects of the extract. In other words, this hyperpolarization in smooth muscle cells leads calcium channels to close, so decreasing the concentration of intracellular calcium and inducing vasodilation.

In summary, the polyphenols found in the extract Murtilla could stimulate the activation of eNOS-NO-GCs pathway, as the vasodilator effects were partially reversed by L-NAME and ODQ. However, it also appears to be an effect on large-conductance calcium-dependent potassium channels (a proposed model is shown in Figure 5). Thus, the data suggest a synergistic effect of the compounds present in the extract leading to decreased tension of the aortic rings.

A study showed that the application of an important phenol, resveratrol, induces the overexpression of eNOS and NO in a concentration-dependent manner between 24 and 72 hours in EA cells, while in HUVEC cells these effects are observed up to 925 hours [48]. Furthermore, the cardioprotective effects of this phenol were investigated* in vivo*. Hypertensive rats previously treated with resveratrol showed decreased oxidative stress, preserved endothelial function, and amelioration of hypertension, preventing uncoupling of eNOS and stimulating the production of NO [49]. Additionally, it has been shown that the exposure of resveratrol (30 *μ*M) to HUVEC-C cells increases reversibly the amplitude of potassium currents, increasing the opening time and decreasing the closing time of LK channels [50]. In the same aspect, Quercetin has been reported as effective vasodilator in isolated pulmonary restoring K_v_ currents [51] and demonstrated in hypertensive humans [52].

Altogether, these findings show evidence that the use of food preparations obtained from fruits and leaves may have beneficial effects in preventing and possibly treating the symptoms of cardiovascular diseases. In this study, we demonstrated that Murtilla fruit extract has significant antioxidant and vasodilator properties. We also proposed mechanistic models to explain the antioxidant and hypotensive effects observed.

## Figures and Tables

**Table 1:** Concentration of phenolic compounds identified in the aqueous fresh extract from the fruits of *Ugni molinae *Turcz. (Murtilla), genotype 14-4 INIA, by high-performance liquid chromatography.

Compound	Retention time (min)	Concentration (*µ*g/mL)
Gallic acid	7.72	0.059 ± 0.022
Catechin	10.07	2.696 ± 0.690
Quercetin-3-*β*-D-glucoside	21.18	0.141 ± 0.086
Myricetin	22.68	0.115 ± 0.001
Quercetin	24.02	0.009 ± 0.001
Kaempferol	25.85	0.014 ± 0.008

## List of Abbreviations

Os: Oxygen species
ROS: Reactive oxygen species
HPLC: High-performance liquid chromatography
GAE: Gallic acid equivalent
HUVEC: Human Umbilical Vein Endothelial Cells
DMEM: Dulbecco's Modified Eagle's Medium
DHE: Dihydroethidium
TYRO37: Tyrode modified solution under constant oxygenation
NO: Nitric oxide
FRAP: Ferric reducing antioxidant power
NRF2: Nuclear factor (erythroid-derived 2)-like 2
ARE: Antioxidant response element
NO: Nitric oxide
eNOS: Endothelial nitric oxide synthase.

## References

[1] Go A. S., Mozaffarian D., Roger V. L. (2014). Heart disease and stroke statistics—2014 update: a report from the American Heart Association. *Circulation*.

[2] Rajendran P., Nandakumar N., Rengarajan T. (2014). Antioxidants and human diseases. *Clinica Chimica Acta*.

[3] Zampetaki A., Dudek K., Mayr M. (2013). Oxidative stress in atherosclerosis: the role of microRNAs in arterial remodeling. *Free Radical Biology and Medicine*.

[4] Aviram M. (2011). Atherosclerosis: cell biology and lipoproteins—inflammation and oxidative stress in atherogenesis: protective role for paraoxonases. *Current Opinion in Lipidology*.

[5] Durmus A., Mentese A., Yilmaz M. (2014). The thrombotic events in polycythemia vera patients may be related to increased oxidative stress. *Medical Principles and Practice*.

[6] Higashi Y., Noma K., Yoshizumi M., Kihara Y. (2009). Endothelial function and oxidative stress in cardiovascular diseases. *Circulation Journal*.

[7] Ciacci C., Russo I., Bucci C. (2014). The kiwi fruit peptide kissper displays anti-inflammatory and anti-oxidant effects in in-vitro and ex-vivo human intestinal models. *Clinical and Experimental Immunology*.

[8] Halvorsen B. L., Carlsen M. H., Phillips K. M. (2006). Content of redox-active compounds (ie, antioxidants) in foods consumed in the United States. *American Journal of Clinical Nutrition*.

[9] Jesus Periago M., García-Alonso J., Jacob K. (2009). Bioactive compounds, folates and antioxidant properties of tomatoes (*Lycopersicum esculentum*) during vine ripening. *International Journal of Food Sciences and Nutrition*.

[10] Dreher M. L., Davenport A. J. (2013). Hass avocado composition and potential health effects. *Critical Reviews in Food Science and Nutrition*.

[11] Tuso P. J., Ismail M. H., Ha B. P., Bartolotto C. (2013). Nutritional update for physicians: plant-based diets. *The Permanente Journal*.

[12] Goufo P., Trindade H. (2014). Rice antioxidants: phenolic acids, flavonoids, anthocyanins, proanthocyanidins, tocopherols, tocotrienols, gamma-oryzanol, and phytic acid. *Food Science & Nutrition*.

[13] Erlund I., Koli R., Alfthan G. (2008). Favorable effects of berry consumption on platelet function, blood pressure, and HDL cholesterol. *American Journal of Clinical Nutrition*.

[14] Scheuermann E., Seguel I., Montenegro A., Bustos R. O., Hormazábal E., Quiroz A. (2008). Evolution of aroma compounds of murtilla fruits (*Ugni molinae* Turcz) during storage. *Journal of the Science of Food and Agriculture*.

[15] Shene C., Reyes A. K., Villarroel M., Sineiro J., Pinelo M., Rubilar M. (2009). Plant location and extraction procedure strongly alter the antimicrobial activity of murta extracts. *European Food Research and Technology*.

[16] Rubilar M., Pinelo M., Ihl M., Scheuermann E., Sineiro J., Nuñez M. J. (2006). Murta leaves (Ugni molinae turcz) as a source of antioxidant polyphenols. *Journal of Agricultural and Food Chemistry*.

[17] Alfaro S., Mutis A., Quiroz A., Seguel I., Scheuermann E. (2014). Effects of drying techniques on murtilla fruit polyphenols and antioxidant activity. *Journal of Food Research*.

[18] Mosmann T. (1983). Rapid colorimetric assay for cellular growth and survival: application to proliferation and cytotoxicity assays. *Journal of Immunological Methods*.

[19] Boess F., Boelsterli U. A. (2002). Luminol as a probe to assess reactive oxygen species production from redox-cycling drugs in cultured hepatocytes. *Toxicology Mechanisms and Methods*.

[20] Drummen G. P. C., van Liebergen L. C. M., Op den Kamp J. A. F., Post J. A. (2002). C11-BODIPY^581/591^, an oxidation-sensitive fluorescent lipid peroxidation probe: (micro)spectroscopic characterization and validation of methodology. *Free Radical Biology and Medicine*.

[21] Wen Y.-D., Wang H., Kho S.-H. (2013). Hydrogen sulfide protects HUVECs against hydrogen peroxide induced mitochondrial dysfunction and oxidative stress. *PLoS ONE*.

[22] Chen S., Tang Y., Qian Y. (2014). Allicin prevents H_2_O_2_-induced apoptosis of HUVECs by inhibiting an oxidative stress pathway. *BMC Complementary and Alternative Medicine*.

[23] Ruiz A., Hermosín-Gutiérrez I., Mardones C. (2010). Polyphenols and antioxidant activity of calafate (*Berberis microphylla*) fruits and other native berries from Southern Chile. *Journal of Agricultural and Food Chemistry*.

[24] Suzuki T., Takagi A., Takahashi M. (2012). Catechin-rich green tea extract increases serum cholesterol levels in normal diet-and high fat diet-fed rats. *BMC Proceedings*.

[25] Muzolf-Panek M., Gliszczyńska-Świgło A., Szymusiak H., Tyrakowska B. (2012). The influence of stereochemistry on the antioxidant properties of catechin epimers. *European Food Research and Technology*.

[26] Augusto T. R., Salinas E. S. S., Alencar S. M., D’Arce M. A. B. R., De Camargo A. C., Vieira T. M. F. D. S. (2015). Phenolic compounds and antioxidant activity of hydroalcoholic extractsof wild and cultivated murtilla (*Ugni molinae* turcz.). *Food Science and Technology*.

[27] Junqueira-Gonçalves M. P., Yáñez L., Morales C., Navarro M., Contreras R. A., Zúñiga G. E. (2015). Isolation and characterization of phenolic compounds and anthocyanins from murta (Ugni molinae Turcz.) fruits. Assessment of antioxidant and antibacterial activity. *Molecules*.

[28] Alfaro S., Mutis A., Palma R., Quiroz A., Seguel I., Scheuermann E. (2013). Influence of genotype and harvest year on polyphenol content and antioxidant activity in murtilla (*Ugni molinae* Turcz) fruit. *Journal of Soil Science and Plant Nutrition*.

[29] Gough D. R., Cotter T. G. (2011). Hydrogen peroxide: a Jekyll and Hyde signalling molecule. *Cell Death and Disease*.

[30] Rumley A. G., Woodward M., Rumley A., Rumley J., Lowe G. D. O. (2004). Plasma lipid peroxides: relationships to cardiovascular risk factors and prevalent cardiovascular disease. *QJM*.

[31] Frankel E. N. (1980). Lipid oxidation. *Progress in Lipid Research*.

[32] Busse R., Fleming I. (1996). Endothelial dysfunction in atherosclerosis. *Journal of Vascular Research*.

[33] Harrison D. G. (1997). Cellular and molecular mechanisms of endothelial cell dysfunction. *The Journal of Clinical Investigation*.

[34] Mayhan W. G. (1989). Impairment of endothelium-dependent dilatation of cerebral arterioles during diabetes mellitus. *American Journal of Physiology-Heart and Circulatory Physiology*.

[35] Katusic Z. S. (1996). Superoxide anion and endothelial regulation of arterial tone. *Free Radical Biology and Medicine*.

[36] Ohara Y., Peterson T. E., Harrison D. G. (1993). Hypercholesterolemia increases endothelial superoxide anion production. *Journal of Clinical Investigation*.

[37] Saw C. L. L., Yang A. Y., Huang M.-T. (2014). Nrf2 null enhances UVB-induced skin inflammation and extracellular matrix damages. *Cell and Bioscience*.

[38] Lee J.-M., Johnson J. A. (2004). An important role of Nrf2-ARE pathway in the cellular defense mechanism. *Journal of Biochemistry and Molecular Biology*.

[39] Dinkova-Kostova A. T., Holtzclaw W. D., Cole R. N. (2002). Direct evidence that sulfhydryl groups of Keap1 are the sensors regulating induction of phase 2 enzymes that protect against carcinogens and oxidants. *Proceedings of the National Academy of Sciences of the United States of America*.

[40] Granado-Serrano A. B., Martín M. A., Bravo L., Goya L., Ramos S. (2012). Quercetin modulates Nrf2 and glutathione-related defenses in HepG2 cells: involvement of p38. *Chemico-Biological Interactions*.

[41] Cheng Y.-T., Wu C.-H., Ho C.-Y., Yen G.-C. (2013). Catechin protects against ketoprofen-induced oxidative damage of the gastric mucosa by up-regulating Nrf2 in vitro and in vivo. *Journal of Nutritional Biochemistry*.

[42] Graham H. N. (1992). Green tea composition, consumption, and polyphenol chemistry. *Preventive Medicine*.

[43] Heim K. E., Tagliaferro A. R., Bobilya D. J. (2002). Flavonoid antioxidants: chemistry, metabolism and structure-activity relationships. *Journal of Nutritional Biochemistry*.

[44] Bhardwaj P., Khanna D. (2013). Green tea catechins: defensive role in cardiovascular disorders. *Chinese Journal of Natural Medicines*.

[45] Angelone T., Pasqua T., Di Majo D. (2011). Distinct signalling mechanisms are involved in the dissimilar myocardial and coronary effects elicited by quercetin and myricetin, two red wine flavonols. *Nutrition, Metabolism and Cardiovascular Diseases*.

[46] Namiki A., Hirata Y., Ishikawa M., Moroi M., Aikawa J., Machii K. (1992). Endothelin-1- and endothelin-3-induced vasorelaxation via common generation of endothelium-derived nitric oxide. *Life Sciences*.

[47] Kochukov M. Y., Balasubramanian A., Abramowitz J., Birnbaumer L., Marrelli S. P. (2014). Activation of endothelial transient receptor potential C3 channel is required for small conductance calcium-activated potassium channel activation and sustained endothelial hyperpolarization and vasodilation of cerebral artery. *Journal of the American Heart Association*.

[48] Wallerath T., Deckert G., Ternes T. (2002). Resveratrol, a polyphenolic phytoalexin present in red wine, enhances expression and activity of endothelial nitric oxide synthase. *Circulation*.

[49] Bhatt S. R., Lokhandwala M. F., Banday A. A. (2011). Resveratrol prevents endothelial nitric oxide synthase uncoupling and attenuates development of hypertension in spontaneously hypertensive rats. *European Journal of Pharmacology*.

[50] Li H.-F., Chen S.-A., Wu S.-N. (2000). Evidence for the stimulatory effect of resveratrol on Ca^2+^-activated K^+^ current in vascular endothelial cells. *Cardiovascular Research*.

[51] Morales-Cano D., Menendez C., Moreno E. (2014). The flavonoid quercetin reverses pulmonary hypertension in rats. *PLoS ONE*.

[52] Edwards R. L., Lyon T., Litwin S. E., Rabovsky A., Symons J. D., Jalili T. (2007). Quercetin reduces blood pressure in hypertensive subjects. *Journal of Nutrition*.

